# Pecto-Intercostal Fascial Plane Block: Effect on the Postoperative Analgesia and Recovery After Off-PUMP Coronary Artery Bypass Surgery

**DOI:** 10.5812/aapm-144344

**Published:** 2024-02-15

**Authors:** Ramy Mahrose, Hany Magdy Fahim, Amr A. Kasem, Mohammed Samy Helmy Sakr, Mohammed Abdelsalam Menshawi

**Affiliations:** 1 Assistant Professor of Anesthesia, Intensive Care and Pain Management, Faculty of Medicine, Ain Shams University, Cairo, Egypt; 2 Lecturer of Anesthesia, Intensive Care and Pain Management, Faculty of Medicine, Ain Shams University, Cairo, Egypt; 3 Lecturer of Radiology, Faculty of Medicine, Tanta University, Tanta, Egypt

**Keywords:** Off-Pump Coronary Artery Bypass Surgery, Pecto-Intercostal Fascial Block, Postoperative Analgesia, Opioid Consumption

## Abstract

**Background:**

Anteromedial chest wall fascial plane blocks may serve as a valuable addition to postoperative multimodal pain management following median sternotomy for cardiothoracic surgeries.

**Objectives:**

This study aimed to evaluate the impact of implementing the pecto-intercostal fascial plane block (PIFB) in patients scheduled for off-pump coronary artery bypass (OPCAB) surgery.

**Methods:**

This randomized controlled study involved 40 adult patients aged 30 to 70 years undergoing OPCAB surgery. They were randomly assigned to two equal groups: Group PI received bilateral ultrasound (US)-guided PIFB with 20 mL of bupivacaine 0.25% with adrenaline 2.5 µg/mL, while group C (control group) received bilateral sham blocks with 20 mL of saline 0.9%. Pain scores in the postoperative period (primary outcome), perioperative analgesic consumption, time until extubation, and discharge from the intensive care unit (ICU) were assessed for both groups.

**Results:**

Postoperative pain scores, both at rest and during coughing, were significantly lower in group PI compared to group C. Group PI required significantly less fentanyl perioperatively and less tramadol for postoperative rescue compared to group C. The duration of postoperative ventilation and time to ICU discharge were significantly shorter in group PI than in group C.

**Conclusions:**

In patients undergoing OPCAB surgery, pre-incisional ultrasound-guided PIFB can be a beneficial and safe component of multimodal pain management. It provides improved postoperative pain control, reduces the need for perioperative opioids, and leads to faster extubation and ICU discharge.

## 1. Background

When comparing on-pump and off-pump coronary artery bypass (OPCAB) surgeries, previous research has indicated that on-pump procedures tend to result in longer periods of postoperative mechanical ventilation and extended stays in the intensive care unit (ICU) and hospital. These differences are often attributed to factors such as aortic manipulation, which triggers a systemic inflammatory response, a higher risk of bleeding, adverse neurological events, and potential kidney dysfunction (1, 2).

In open cardiac surgeries, the median sternotomy incision is a major source of postoperative pain. Traditional approaches have involved the administration of high doses of opioids to achieve adequate pain management (3). However, a multimodal approach has gained popularity, involving the use of regional or neuraxial techniques and an increased reliance on non-opioid analgesics to effectively control postoperative pain while minimizing opioid-related side effects (4).

While techniques like paravertebral block (PVB) and thoracic epidural analgesia (TEA) have been effective in providing postoperative analgesia following sternotomy (5, 6), they come with their own set of complications and limitations, particularly in cases where systemic anticoagulation is required during cardiac surgery (7).

Interfascial thoracic wall plane blocks have emerged as a valuable component of multimodal analgesia after cardiac surgery. The pecto-intercostal fascial plane block (PIFB) is an ultrasound-guided parasternal technique that involves the injection of local anesthetic (LA) into the plane between the intercostal muscles and the pectoralis major muscle. This technique aims to block the anterior cutaneous branches of the thoracic nerves, specifically T2-T6, which innervate the anteromedial chest wall, including the sternum (8-10).

## 2. Objectives

The objective of this study was to assess the impact of implementing PIFB in patients undergoing OPCAB surgery, specifically in terms of the quality of postoperative pain control, perioperative analgesic requirements, time to extubation, and discharge from the ICU.

## 3. Methods

After obtaining approval from the research ethics committee at the Faculty of Medicine, Ain Shams University (FMASU R 34/2023) and registering the study on ClinicalTrials.gov (NCT05774249), we conducted a randomized, prospective, double-blind study between March 2023 and July 2023 at Ain Shams University hospitals.

Our study involved forty adult patients, aged between 30 and 70 years, who had undergone OPCAB surgery and had a New York Heart Association (NYHA) Functional Classification of 1 - 3. These patients provided written informed consent. They were randomly assigned to one of two equal groups: The control group (group C) and the PIFB group (group PI).

### 3.1. Exclusion Criteria

Exclusion criteria encompassed individuals with unsuitable targets for surgery (such as intramyocardial or diffusely diseased vessels), those undergoing combined coronary artery bypass grafting (CABG) with valve replacement, redo CABG, individuals with poor myocardial contractility (ejection fraction ≤ 35%), those with preexisting hemodynamic instability requiring inotropes or intra-aortic balloon pump insertion, cases of intraoperative conversion to on-pump CABG, postoperative complications necessitating re-exploration for surgical issues or resulting in death, severe chronic obstructive pulmonary disease, myasthenia and myopathies, morbid obesity, cognitive dysfunction, or difficulty in communication, as well as individuals with contraindications to the block procedure, such as allergies to LAs or patient refusal.

### 3.2. Study Procedure

A thorough patient assessment was conducted the day before the surgery. This assessment included checking and correcting serum electrolytes, including potassium (K) and magnesium. Perioperatively, beta-blockers were continued, while angiotensin-converting enzyme inhibitors and antiplatelet drugs were discontinued 24 hours and 5 days before the surgery, respectively. On the night before the surgery, the patient received a 30 mg tablet of lansoprazole and a 0.5 mg tablet of alprazolam.

On the day of the surgery, it was ensured that patients were fasting. Upon their arrival in the operating theater, various monitoring measures were established, including five-lead electrocardiography (ECG), pulse oximetry, and non-invasive blood pressure monitoring. Additionally, a bispectral (BIS) index monitor and peripheral nerve stimulator were applied. Patients were premedicated with intravenous 3rd generation cephalosporin, ondansetron 8 mg, midazolam (0.02 - 0.03 mg/kg), and fentanyl (50 - 100 mcg). Under LA infiltration, either a radial or femoral arterial cannula was inserted for invasive arterial blood pressure monitoring. Baseline measurements of arterial blood gases (ABGs) and activated clotting time (ACT) were performed.

For the induction of general anesthesia, intravenous propofol was titrated to achieve a BIS level below 60. Subsequently, fentanyl (5 µg/kg) and cisatracurium (0.15 mg/kg) were administered. An endotracheal tube was inserted, and mechanical ventilation was initiated. Central venous cannulation was performed under ultrasound (US) guidance, and probes for nasopharyngeal temperature and transesophageal echocardiography (TEE) were inserted.

**Figure 1.**
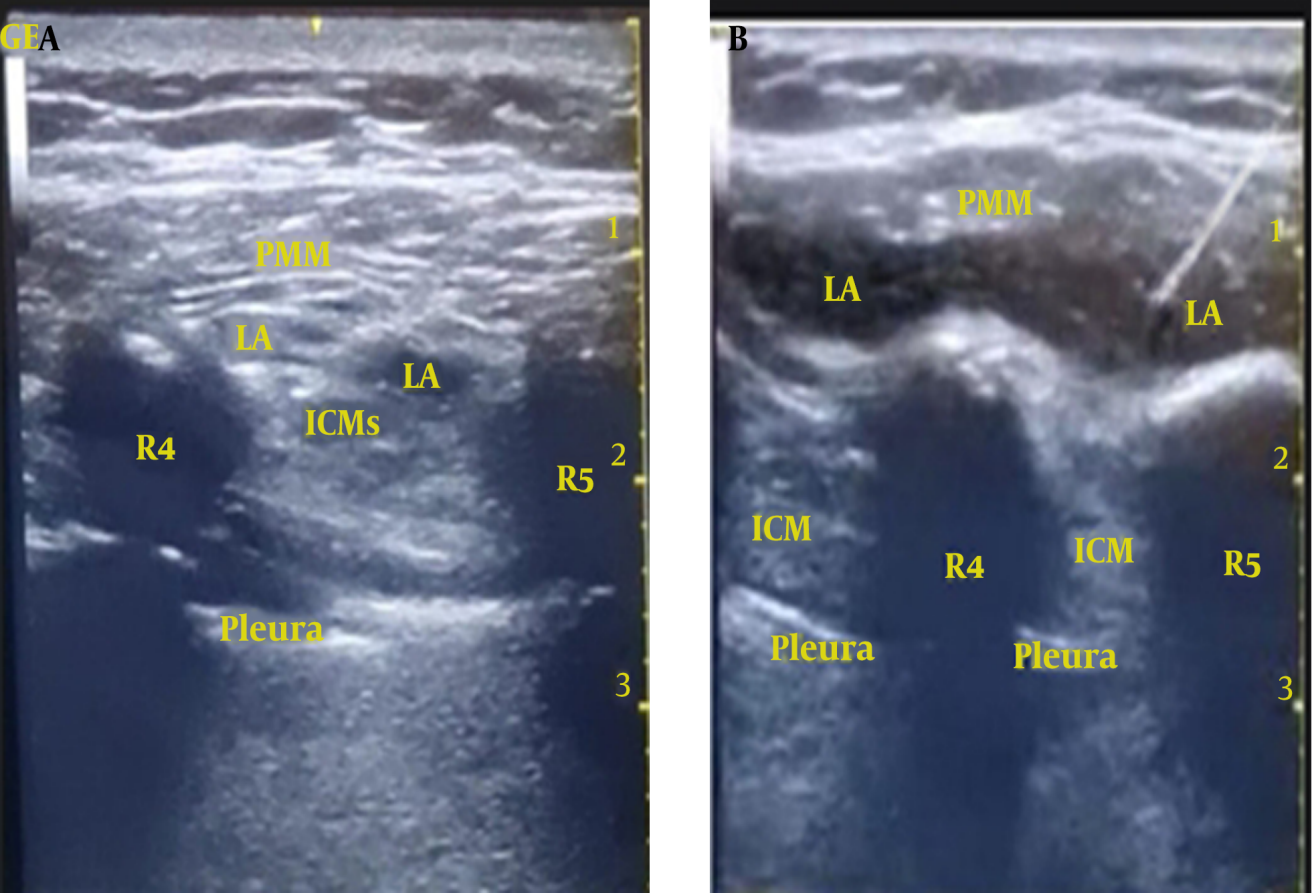
Ultrasound-guided pecto-intercostal fascial plane block (PIFB). A, the local anesthetic (LA) was deposited in the interfascial plane between the pectoralis major and the intercostal muscles; B, LA spread in the targeted pecto-intercostal fascial plane. R4: 4th rib; R5: 5th rib; PMM, pectoralis major muscle; ICM, intercostal muscles.

While patients were positioned in the supine posture, an experienced anesthesiologist, who conducted the parasternal blocks for all patients, initiated a parasagittal US scan (2 cm lateral to the sternum) from top to bottom using a high-frequency linear US probe (5 - 13 MHz). The ribs (evident as a hyperechoic curved line with a shadow underneath) were identified. At the level of the fourth intercostal space, an echogenic 100 mm 20 G block needle was introduced in a caudocephalic direction using an in-plane technique, targeting the plane between the pectoralis major muscle and intercostal muscles. To confirm the correct needle tip placement, hydrodissection was performed using 2 - 3 mL of 0.9% saline. Following a negative check for blood aspiration, 20 mL of 0.25% bupivacaine with 2.5 µg/mL adrenaline was injected incrementally (1 mL every 5 seconds) in group PI, with the needle progressively advanced further into the fascial plane (Figure 1A). The real-time US scanning confirmed the spread of the LA in the target plane in a cranio-caudal direction (Figure 1B). The same procedure was repeated on the opposite side of the sternum. In group C (the control group), a sham block was performed using an equivalent volume of normal saline 0.9% bilaterally.

Surgery commenced 15 minutes after the block was administered. Both the anesthesiologists responsible for data collection, the surgeons, and the ICU staff were unaware of the type of injectate received by the patient. Anesthesia was maintained with sevoflurane to maintain the BIS index within the range of 40 to 60. Incremental doses of fentanyl (1 µg/kg) were administered to keep hemodynamics within 20% of baseline values, along with cisatracurium (0.03 mg/kg) boluses as per train of four (TOF) monitoring. Regular checks of intraoperative ABG were conducted, and serum K levels were maintained within the range of 4 - 4.5 mmol/L.

To achieve a target-ACT of 250 - 300 seconds, patients received heparin at a dose of 150 - 200 IU/kg shortly before the conclusion of the left internal mammary artery (LIMA) harvesting procedure. All patients underwent OPCAB surgery performed by the same cardiothoracic surgery team. The left anterior descending artery was revascularized using the LIMA, while all other grafts were saphenous venous grafts. During grafting, an octopus stabilization system was employed.

Intraoperative hemodynamic fluctuations occurring during the revascularization process were addressed based on transesophageal TEE findings. This involved actions such as optimizing preload through fluid boluses, adjusting the position of the myocardium within the stabilizing devices, and, if necessary, administering vasopressor infusions (such as phenylephrine or norepinephrine) with the goal of maintaining a mean arterial blood pressure of at least 70 mmHg. Intraoperative arrhythmias were managed through the use of antiarrhythmic medications or, when required, direct current (DC) shock. Following the completion of vascular anastomoses, a TEE examination was conducted to assess regional myocardial contractility and identify any wall motion abnormalities. After confirming the proper functioning of grafts, the effects of heparin were reversed using protamine sulfate at a rate of 1 mg per 100 IU of heparin. Chest wall closure was initiated after ensuring sufficient hemostasis. Additionally, a 10 mL solution of 0.25% bupivacaine with 2.5 mcg/mL adrenaline was subcutaneously infiltrated around the exit site of mediastinal drains.

After the conclusion of surgery, patients were transferred to the ICU under the supervision of the responsible anesthesiologist. Postoperative ventilation was continued with continuous monitoring of hemodynamics by a specialized nurse and oversight from an ICU specialist. A range of laboratory tests, including ABG, complete blood count, serum electrolytes, coagulation profile, and serial cardiac enzyme measurements, was conducted, and any abnormalities were addressed accordingly. Furthermore, ECGs and chest X-rays were performed as necessary. The mediastinal drains were monitored to assess the amount of blood loss.

The postoperative pain management plan involves several components:

(1) Intravenous paracetamol (1 g/100 mL) was administered once patients arrived in the ICU and repeated every 6 hours.

(2) Intravenous patient-controlled analgesia (PCA) was implemented using IV fentanyl (20 µg/mL) with a bolus dose of 1 mL and a lockout interval of 15 minutes. There was no continuous basal infusion rate. Initially, this was managed through nurse-controlled analgesia (NCA) before extubation to maintain hemodynamics within 20% of baseline values.

(3) After extubation, patients themselves controlled the IV PCA for pain management.

(4) If a patient requested additional pain relief or if the visual analogue scale (VAS) score reached 4 or higher, despite the above measures, rescue IV tramadol at a dose of 1 mg/kg was administered. The daily maximum dose of tramadol was limited to 300 mg/day.

Patients were regularly evaluated to determine their suitability for extubation based on the following criteria:

- They needed to be conscious and have stable hemodynamics with minimal or no reliance on pharmacological circulatory support, such as inotropes or vasopressors.

- Adequate hemostasis was required, with minimal blood loss through chest drains (less than 100 mL/h) and no indications of cardiac tamponade.

- Satisfactory ABG results and normal serum electrolyte levels.

- Maintenance of normal body temperature (normothermia).

Once these criteria were met, the process of weaning from mechanical ventilation would commence, followed by extubation. Oxygen supplementation was provided through a facemask, and patients were closely monitored for adequate respiratory efforts, with ABG analysis performed as necessary. Patients were encouraged to engage in deep breathing exercises, coughing, and early mobilization with the assistance of a responsible nurse and an attending physiotherapist.

### 3.3. Outcome Measurements

The primary outcome measured in this study was the postoperative VAS scores. These scores were recorded at various time points, including immediately after patient extubation and at 1 hour, 2 hours, 4 hours, 8 hours, 12 hours, 18 hours, and 24 hours post-extubation. Additionally, several secondary outcomes were assessed:

- The cumulative consumption of fentanyl, both during the surgery and in the 24 hours following the surgery.

- The number of patients in each group who required rescue tramadol analgesic during the 24 hours following extubation.

- The time it took for patients to be extubated after the surgery and the total duration of their stay in the ICU.

- Incidence and recording of postoperative complications in both groups.

- Patient satisfaction regarding the quality of postoperative analgesia, measured using a Likert scale. This scale ranged from 1 (very dissatisfied) to 5 (very satisfied) and was administered before patients were discharged from the ICU (11).

### 3.4. Sample Size

The sample size for the study was determined using the PASS program for sample size calculation. A previous study by Kumar et al. (12) served as a reference, indicating that postoperative pain scores were significantly lower in the intervention group compared to the control group. The sample size of 40 patients (20 patients in each group) was chosen to ensure a 99.9% power to detect differences in means between the two groups. This calculation considered a possible 20% dropout rate and used a significance level (alpha) of 0.05 in a two-sided, two-sample, unequal variance z-test. The population mean difference (μ1 - μ2) was assumed to be 1, with standard deviations of 0.5 for both group 1 and group 2.

### 3.5. Statistical Analysis

All the data collected from the participants were organized into tables and subjected to statistical analysis using the SPSS computer software version 18. For quantitative parametric variables, we presented the results as mean values along with their standard deviations. Quantitative non-parametric variables were expressed as medians along with their interquartile ranges (IQR). To compare quantitative parametric variables between the study groups, we utilized an unpaired student's *t*-test. Meanwhile, the Mann-Whitney U test was employed for comparing quantitative non-parametric variables. Categorical variables were presented as the number of patients and percentages, and we compared them using either the chi-square test or Fisher’s exact test. A P- value of less than 0.05 was considered as indicative of statistical significance.

## 4. Results

**Figure 2.**
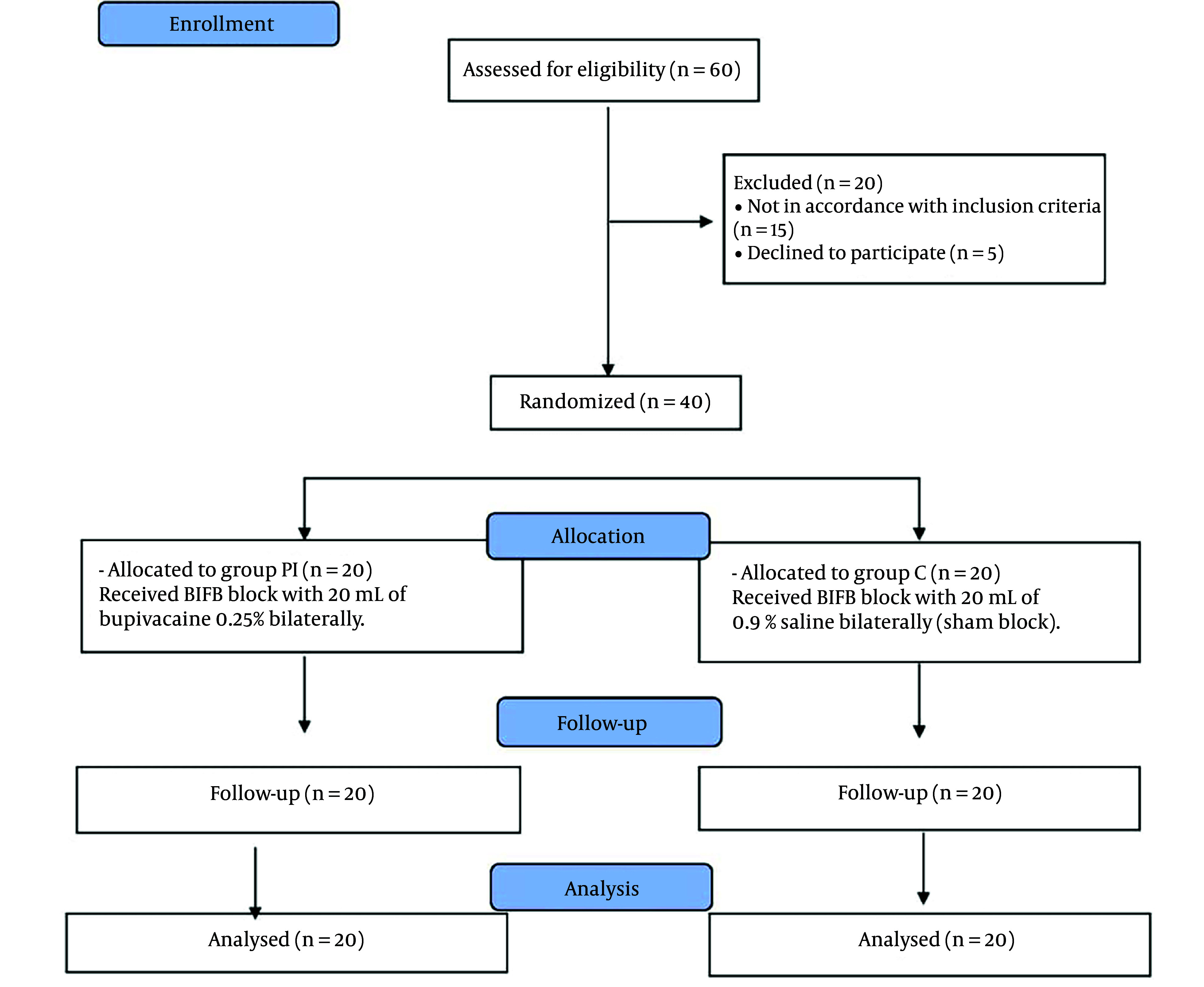
Consort flow diagram of the study stages

As depicted in Figure 2, a total of 60 patients initially slated for OPCAB surgery were screened for eligibility in this study. Among them, 20 patients were excluded: 5 declined to participate, and 15 did not meet the inclusion criteria. Consequently, 40 patients were successfully enrolled and randomized into two equally sized study groups: Group C (control group) and group PI (pesto-intercostal fascial plane block). These participants were closely monitored throughout the study period, and no dropouts or fatalities occurred.

The demographic characteristics of the study patients, along with their underlying medical conditions (Table 1) and details regarding the surgical procedures (Table 2), showed no statistically significant differences between the two groups (P > 0.05).

**Table 1.** Patients’ Demographic Data and Underlying Comorbidities ^a^

	Group C (n = 20)	Group PI (n = 20)	P-Value
**Age (y)**	56.4 ± 7.77	54.15 ± 8.45	0.386
**Sex **			0.723
Male	14	15	
Female	6	5	
**Weight (kg)**	80.40 ± 11.46	77.35 ± 10.47	0.385
**Height (cm)**	168.5 ± 8.46	166.95 ± 6.27	0.525
**Smoking**	11 (55)	9 (45)	0.527
**Hypertensive **	15 (75)	14 (70)	0.723
**Diabetes mellitus **	8 (40)	9 (45)	0.749
**Dyslipidemia**	15 (75)	12 (60)	0.311
**Chronic obstructive pulmonary disease**	2 (10)	2 (10)	1
**Previous PCI**	1 (5)	3 (15)	0.605
**Ejection fraction **	54.4 ± 7.53	53.3 ± 8.59	0.669
**NYHA class**			0.762
I	6 (30)	4 (20)	
II	9 (45)	10 (50)	
III	5 (25)	6 (30)	

Abbreviations: PCI, percutaneous coronary intervention; NYHA, New York Heart Association.

^a^ Values are expressed as mean ± SD or No. (%).

**Table 2.** Operative Details in Study Groups ^a^

	Group C (n = 20)	Group PI (n = 20)	P-Value
**Number of coronary vessels grafted**			0.944
One vessel graft	2 (10)	2 (10)	
Two vessel grafts	8 (40)	7 (35)	
Three vessel grafts	10 (50)	11 (55)	
**Surgical duration**	187.04 ± 33.02	183.8 ± 25.74	0.731

^a^ Values are expressed as mean ± SD or No. (%).

**Figure 3.**
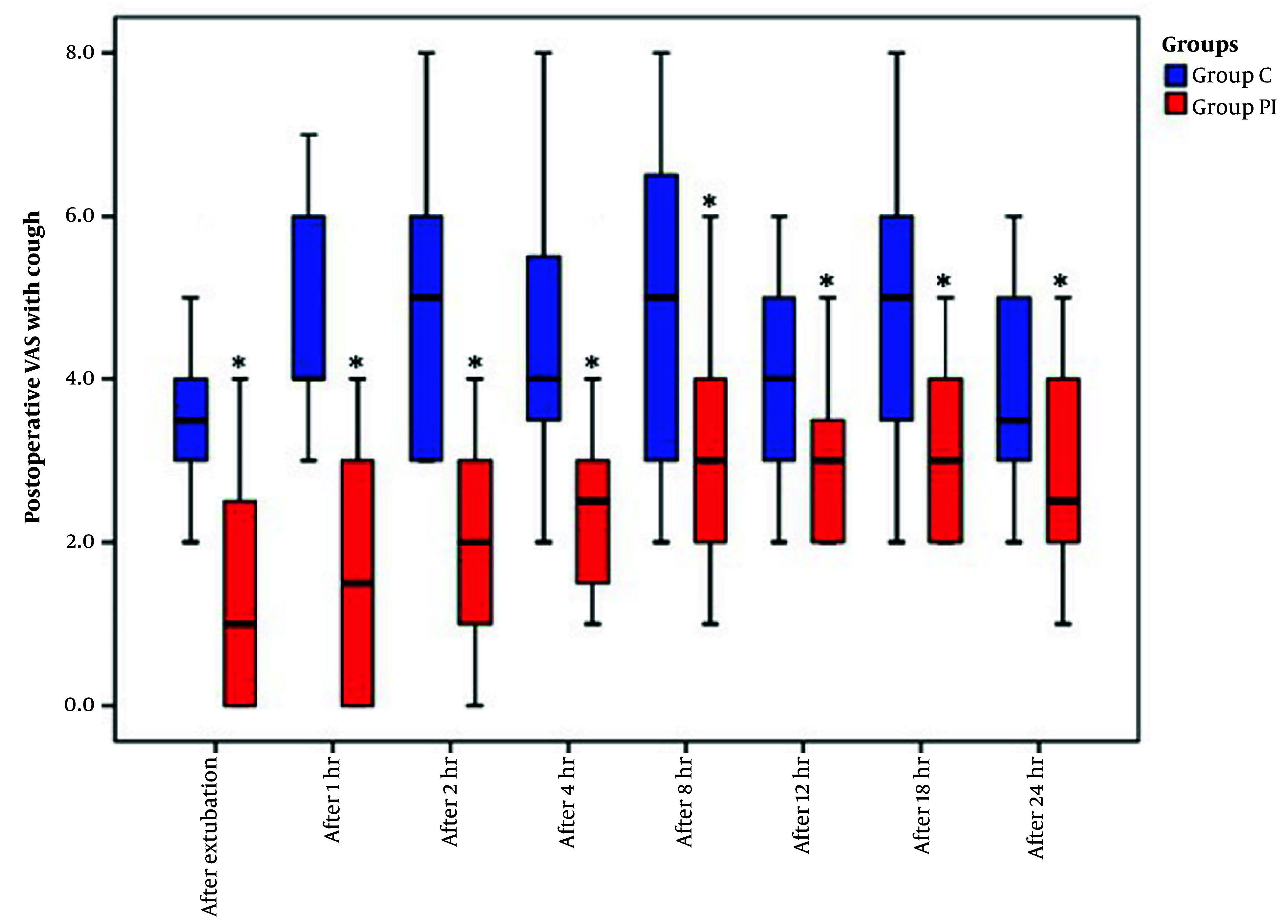
Visual analogue scale (VAS) scores at rest in the study groups. Data are reported as median (IQR). *: Intergroup significant difference.

**Figure 4.**
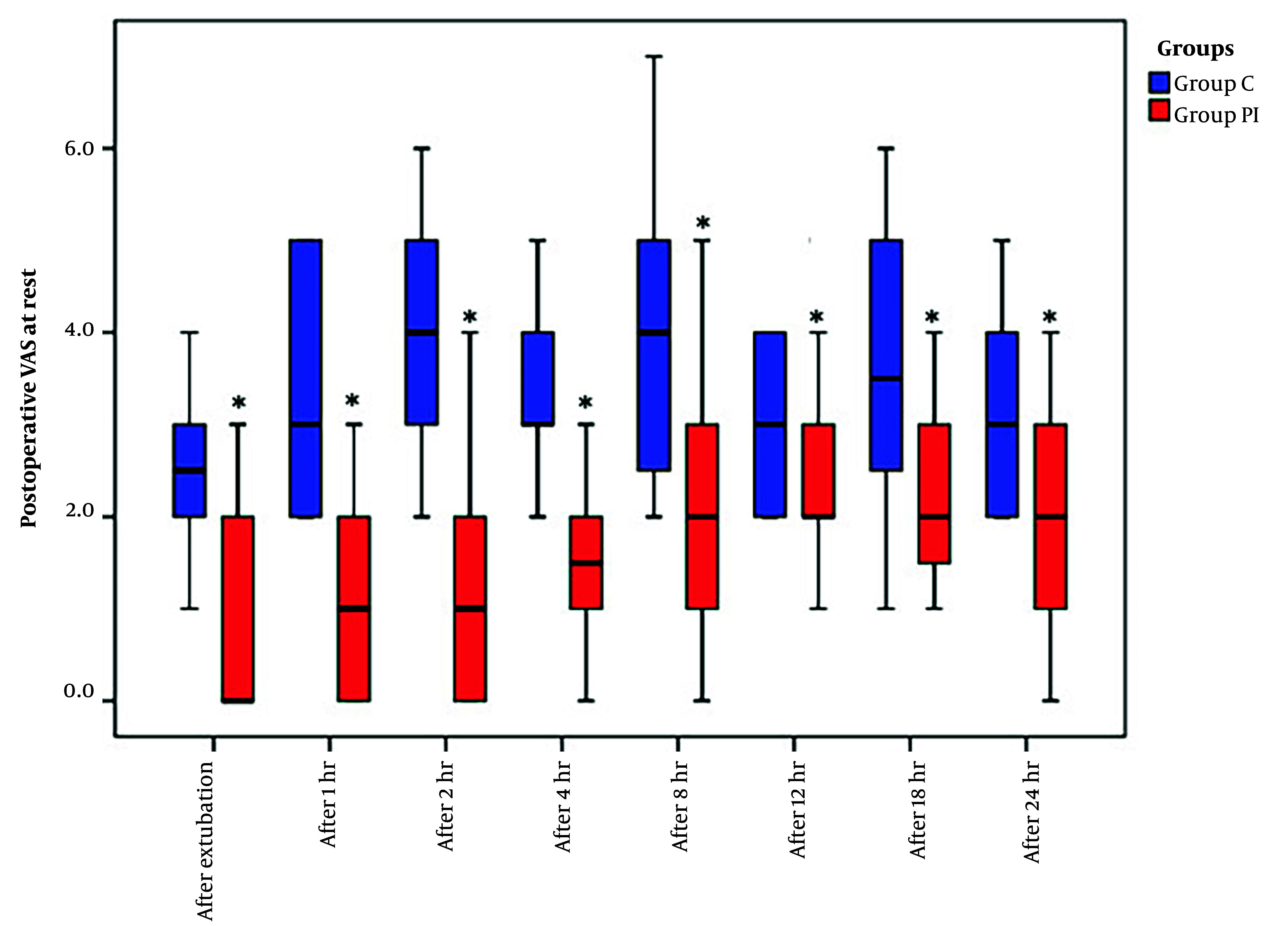
Visual analogue scale (VAS) scores with cough in the study groups. Data are reported as median (IQR). *: Indicate intergroup significant difference.

The VAS scores, recorded both at rest and during coughing over the 24 hours following extubation, exhibited a significant reduction in group PI compared to group C (Figures 3 and 4). 

In terms of fentanyl consumption, both intraoperatively and postoperatively, group PI demonstrated a noteworthy reduction when compared to group C (P < 0.001) (Table 3). Moreover, the number of patients requiring rescue tramadol analgesia was significantly lower in group PI compared to group C (P < 0.05) (Table 3). 

**Table 3.** Perioperative Analgesic Consumption, Time to Extubation, and Intensive Care Unit Discharge ^a^

	Group C (n = 20)	Group PI (n = 20)	P-Value
**Total intraoperative fentanyl consumption (μg)**	849.7 ± 157.58	573.3 ± 108.76 ^b^	< 0.001
**Fentanyl consumption for 24 h postoperatively**	426 ± 84.40	191 ± 41.22 ^b^	< 0.001
**Number of patients needed rescue tramadol**	13 (65)	4 (20) ^b^	0.003
**Time to extubation (min)**	371.30 ± 124.95	196.25 ± 76.83 ^b^	< 0.001
**Time to ICU discharge (h)**	46.5 ± 14.54	36.35 ± 8.94 ^b^	0.011

^a^ Values are expressed as mean ± SD or No. (%).

^b^ Intergroup significant difference.

The time required for extubation following the conclusion of surgery was significantly shorter in group PI compared to group C (P < 0.001). Similarly, the total duration of ICU stay was significantly shorter in group PI compared to group C (P < 0.05) (Table 3). 

Regarding patient satisfaction with postoperative pain management, the scores, presented as a median (IQR), were significantly higher in group PI [5 (4 - 5)] compared to group C [4 (3 - 4)] (P < 0.001).

During the postoperative ICU stay, no significant differences were observed between the two study groups in terms of the incidence of postoperative complications (Table 4). There were no patients in either group who required surgical re-exploration. The incidence of postoperative arrhythmias and the need for circulatory support did not show a significant difference between the two groups (P > 0.05). The PIFB procedure was successfully performed in all patients, and no complications related to it were reported. Opioid-related adverse events were more frequent in group C than in group PI, but the difference was not statistically significant (P > 0.05).

**Table 4.** Postoperative Complications ^a^

	Group C (n = 20)	Group PI (n = 20)	P-Value
**Postoperative complications**			
Bleeding needing surgical re-exploration	0 (0.0)	0 (0.0)	-
Demand for circulatory support (inotropes/vasopressor)	5 (25)	3 (15)	0.694
Postoperative arrhythmia	4 (20)	4 (20)	1
Nausea	7 (35)	3 (15)	0.273
Vomiting	3 (15)	1 (5)	0.605
Pruritus	4 (20)	2 (10)	0.661
Respiratory depression	0 (0.0)	0 (0.0)	-
**Block related complications**			
LA toxicity	0 (0.0)	0 (0.0)	-
Pneumothorax	0 (0.0)	0 (0.0)	-

^a^ Values are expressed as No. (%).

## 5. Discussion

Postoperative pain following sternotomy for cardiac surgery is typically moderate to severe. Traditional approaches that rely on high-dose opioids for effective postoperative pain relief can have several drawbacks, including confusion, respiratory depression, nausea, vomiting, ileus, tolerance, hyperalgesia, and immune suppression (13, 14).

Although neuraxial techniques are effective for postoperative pain management after cardiac surgery, their use is often limited in situations involving intraoperative anticoagulation and the risk of epidural hematoma (15, 16). Advances in ultrasound-guided techniques have led to the development of various thoracic wall plane blocks as part of multimodal postoperative analgesia after sternotomy. These include anterolateral chest wall plane blocks (such as pectoral nerve blocks and serratus anterior plane block) and posterior chest wall plane blocks (such as erector spinae plane block and retrolaminar block) (16-21). However, these blocks may not consistently provide a complete blockade of the anterior branches of the thoracic nerves (22-25).

The anteromedial chest wall fascial plane blocks, which include PIFB and the transverse thoracic muscle plane block (TTPB), are capable of effectively blocking the anterior branches of intercostal nerves T2-T6 (8-10). In our study, we assessed the impact of PIFB as part of a multimodal analgesia approach for patients undergoing OPCAB surgeries. Our findings demonstrated a significant reduction in postoperative pain scores, both at rest and during coughing, for up to 24 hours following extubation. Additionally, there was a significant decrease in perioperative fentanyl consumption in group PI when compared to group C.

These results align with those of previous studies conducted by Zhang et al. (26, 27), which investigated the effect of PIFB on perioperative pain control in adult and pediatric patients undergoing sternotomy for cardiac surgery. In both cases, there was a notable decrease in postoperative pain scores over the first 24 hours after extubation, along with a substantial reduction in opioid consumption among patients who received the block compared to those in the control group.

Two other randomized controlled trials conducted by Kumar et al. (12) and Khera et al. (28) evaluated the efficacy of PIFB as part of postoperative multimodal analgesia for cardiac surgery patients. Both studies reported significantly lower postoperative pain scores in the groups that received the block. While there was a trend towards reduced postoperative opioid consumption in both studies, statistical significance was achieved in only one of them (12).

The effectiveness of PIFB when administered post-incisional after surgery has been demonstrated in previous research (12, 28). In our study, PIFB was performed after anesthesia induction and prior to the start of surgery, serving as preemptive analgesia. This approach offers the advantage of preventing peripheral and central sensitization that can occur as a result of surgical tissue trauma, potentially exacerbating postoperative pain (29). Additionally, it can mitigate the intraoperative noxious stimuli commonly encountered during cardiac surgery, such as skin incision, sternotomy, sternal retraction, and wiring. These stimuli carry the risk of causing significant intraoperative hemodynamic fluctuations in patients with preexisting cardiac ischemia (30). Our results revealed a significantly reduced intraoperative demand for fentanyl, titrated to maintain hemodynamic stability within 20% of baseline values, in group PI compared to group C. Similarly, Bloc et al. (31) reported a lower requirement for intraoperative remifentanil to maintain hemodynamic stability during CABG surgeries in the parasternal block group when compared to the control group.

In this study, the significant reduction in both intraoperative and postoperative fentanyl usage, along with the superior pain relief provided in the PIFB group, likely contributed to the significantly faster extubation and earlier discharge from the ICU in comparison to the control group (group C). These findings align with the results reported by Zhang et al. (26, 27) and Thomas et al. (32). Pecto-intercostal fascial plane block has also been successfully employed for rescue analgesia in a patient who had extensive chest wall trauma and a sternal body fracture following an inadequate thoracic epidural block, resulting in marked improvement in ventilatory function (33). Additionally, it has been used to manage pain and facilitate weaning in a critically ill patient with retractable ribcage pain at the site of endothoracic drainage (34).

Importantly, no complications related to the block were observed in either of the study groups. Given that the study patients underwent OPCAB procedures, the decision was made to employ US-guided PIFB instead of TTPB for the block on the parasternal intercostal nerves. This choice aimed to minimize the potential risk of injury to the LIMA, which is often harvested for coronary grafting during these surgeries. Additionally, PIFB is relatively more superficial, which reduces the risk of pleural injury and pneumothorax (35-37). Although the incidence of opioid-related adverse events was lower in group PI compared to group C, this difference did not reach statistical significance. However, it could be attributed to the significantly lower perioperative opioid consumption in the PIFB group compared to the control group (group C).

### 5.1. Limitations

There are certain limitations to this study. Firstly, even though sternotomy is a major source of postoperative pain following OPCAB surgeries, postoperative pain is multifaceted, encompassing visceral origins and graft harvesting sites. In this study, we relied on a multimodal systemic approach to manage postoperative pain, combining regular paracetamol and opioid use through PCA, along with PIFB, to address pain originating from multiple sources. 

Secondly, we were unable to assess the specific dermatomal sensory blockade of the block, as it was administered after anesthesia induction. This was done to minimize additional stress on patients with preexisting ischemic heart disease. Instead, we ensured proper placement of the block needle in the targeted plane under US guidance, further confirmed by saline hydro dissection and the observed spread of the injectate within the intended plane under US guidance.

Thirdly, we employed a pre-incisional single-shot PIFB block. However, the insertion of a catheter may offer extended analgesia following sternotomy, and this approach should be subject to further evaluation.

Lastly, it's important to note that this study was conducted at a single center with a relatively small sample size, focusing exclusively on OPCAB surgery. Therefore, future investigations should consider a larger and more diverse population sample size, as well as include other types of cardiothoracic surgeries involving sternotomy for a more comprehensive assessment of the analgesic effects of PIFB block.

### 5.2. Conclusions 

For patients undergoing OPCAB surgery, pre-incisional ultrasound-guided PIFB could be a useful and safe component of multimodal analgesia via better postoperative pain control and its perioperative opioid sparing effect with faster extubation and ICU discharge.

## Data Availability

The data sets generated during and/or analyzed during the current study are available from the corresponding author upon reasonable request.

## Clinical Trial Registration Code:

The study was registered at the ClinicalTrials.gov (Clinical Trial ID: NCT05774249) on March 20, 2023.
